# Application of Microwave Irradiation and Heat to Improve Gliadin Detection and Ricin ELISA Throughput with Food Samples 

**DOI:** 10.3390/toxins7062135

**Published:** 2015-06-11

**Authors:** Eric A. E. Garber, Joseph Thole

**Affiliations:** 1 Office of Regulatory Science, Center for Food Safety and Applied Nutrition, Food and Drug Administration, College Park, MD 20740, USA; 2 Joint Institute for Food Safety and Applied Nutrition (JIFSAN), University of Maryland, College Park, MD 20742, USA; E-Mail: jthole@terpmail.umd.edu

**Keywords:** microwave, ELISA, ricin, gluten

## Abstract

The utility of microwave irradiation to accelerate the onset of equilibrium and improve ELISA performance was examined using ELISAs for the detection of the plant toxin ricin and gliadin. The ricin ELISA normally requires several one hour incubations at 37 °C, a total assay time of approximately five hours, and employs a complex buffer containing PBS, Tween-20^®^, and non-fat milk. Different energy levels and pulse designs were compared to the use of abbreviated incubation times at 37 °C for the detection of ricin in food. The use of microwave irradiation had no significant advantage over the application of heat using an oven incubator and performed worse with some foods. In contrast, a gliadin ELISA that relied on 30 min incubation steps at room temperature and a salt-based buffer performed better upon irradiation but also displayed improvement upon incubating the microtiter plate at 37 °C. Whether microwave irradiation was advantageous compared to incubation in an oven was inconclusive. However, by abbreviating the incubation time of the ricin ELISA, it was possible to cut the assay time to less than 2 hours and still display LOD values < 10 ppb and recoveries of 78%–98%.

## 1. Introduction

Maximum sensitivity and robustness in a binding assay occurs when steady-state equilibrium is achieved. However, achieving equilibrium can be time consuming. Thus, to increase throughput, many binding assays do not pursue equilibrium but instead choose incubation (reaction) times that result in sufficient sensitivity and an acceptable variance. Alternatively, many method developers incorporate protocols designed to accelerate the onset of steady-state equilibrium [1].

One of the most commonly used approaches to accelerate the onset of equilibrium and the binding process to enhance the performance of binding–based assays involves the application of heat. However, too much heat can denature proteinaceous ligands or cause changes in the thermodynamic properties of the reactants and product complexes that may nullify the reliability of the assay. This is especially a concern when dealing with complex food samples. Another approach to hasten the onset of equilibrium includes the use of non-ionizing radiation (*i.e.*, microwave and acoustic). Microwave radiation has shown promise due to the ability to control its application. It is possible to quickly heat samples with high efficiency with the use of microwave absorbing containers and sample blocks enhancing the process [2,3,4,5,6]. Similarly, microwave transparent materials can be used; by using such materials, heating can be minimized and the energy focused on macromolecular motion and conformational rearrangement [7,8,9]. This was proposed by Bohr and Bohr [8,9] in which the application of external electromagnetic radiation at 2.45 GHz, supposedly close to the frequency of intrinsic torsional modes of β-lactoglobulin, enhanced the rate of conformational refolding and equilibration. The effect on bond motion should be distinguished from structural changes for which molecular modeling calculations indicate that commonly employed microwave instruments do not provide sufficient energy [10,11]. However, the increase in the energetics of critical bonds may serve to increase the propensity to achieve states conducive to productive interactions involving macromolecules, such as antigen-antibody binding. Thus, microwave irradiation may provide a unique non-thermal approach to enhance binding assays by specifically targeting the macromolecular bonds and ultimately facilitating their reactivity [12,13]. The application of microwave irradiation to enhance ELISA performance has been reported [13,14,15,16]. Optimization of microwave exposure has focused on varying radiation exposure time and power with units constructed with various feedback controls [2,17,18].

The ricin ELISA developed by Tetracore, Inc. is uniquely robust with excellent sensitivity and has been extensively validated for use with food samples [19]. However, this ELISA requires approximately 5 h due to the reliance on multiple one hour incubation steps at 37 °C to achieve steady-state equilibrium. In contrast, the R5 sandwich ELISA, which was endorsed as a type 1 method for the detection of gluten by the Codex Committee on Methods on Analysis and Sampling (CCMAS) [20], relies on the use of 30 min incubation steps at room temperature.

A method that decreases assay time with minimal impact on ELISA performance increases the utility of the method, especially when throughput is critical. Whether microwave irradiation offers an advantage over the use of standard incubators was examined for a ricin sandwich ELISA with five food matrices, a chocolate-hazelnut spread, whole wheat pita bread, orange juice with pulp, cola-flavored soda, and buffered dilute nonfat milk. Since, heating above 37 °C can be problematic with some food products, it was important to employ power settings and pulse patterns that did not overheat the samples.

## 2. Results and Discussion

### 2.1. Gliadin ELISA

The effects of microwave irradiation on the performance of the RIDASCREEN^®^ Gliadin R5 sandwich ELISA, using gliadin standards in buffered solutions, illustrated the complexity and potential advantage of microwave irradiation. The ELISA protocol specified the use of 30 min incubation steps at room temperature (30 RT) for the sample, enzyme-conjugate, and substrate. Following the recommended procedure, the ELISA displayed an LOD value of 1.1 ng/mL. Changing the sample and enzyme-conjugate incubation steps to entail either a) microwave irradiation at 300 W using a pulse pattern cycling ten times between one min of irradiation and 33 s of no irradiation [(1 min_on_ − 33 s_off_)_9_ − 1 min_on_, abbreviated as 1–33] with maximum peak temperature of < 37 °C, b) 10 min at 37 °C followed by 5 min at 20 °C (10–5), or c) 30 min at 37 °C resulted in LOD values of 1.5, 2.5, and 1.5 ng/mL, respectively. Interestingly, the responses (OD 450 nm) observed with the 40 ng/mL gliadin standard were substantially different for the different incubation protocols with the 30RT, 1–33, 10–5, and 30 min at 37 °C displaying responses of 0.58, 0.41, 0.29, and 0.78, respectively. The use of microwave irradiation for a total of 10 min and 5 min of cooling, substantially improved the performance of the ELISA (LOD and response) *vs.* incubation using an oven for ten min followed by 5 min of cooling. However, incubating at 20 °C or 37 °C for 30 min displayed comparable LOD values and improved responses. Overall, the improvement in the magnitude of the responses were characteristic of the ELISA not achieving steady-state equilibrium binding, but sufficiently optimized that the LOD values were comparable. Thus, microwave irradiation appeared to provide an advantage towards the ELISA, though not necessarily sufficient to justify its use.

### 2.2. Ricin ELISA

To better evaluate the effectiveness of microwave irradiation, a ricin ELISA was evaluated. The ricin ELISA has been extensively validated and is extremely sensitive and robust in part due to its incubating the samples and detector antibodies (primary and secondary-conjugated to HRP) under conditions that achieve steady-state equilibrium. To achieve steady-state equilibrium, the assay employs three one hour incubations at 37 °C, with a total assay time of approximately 5 h.

**Figure 1:**
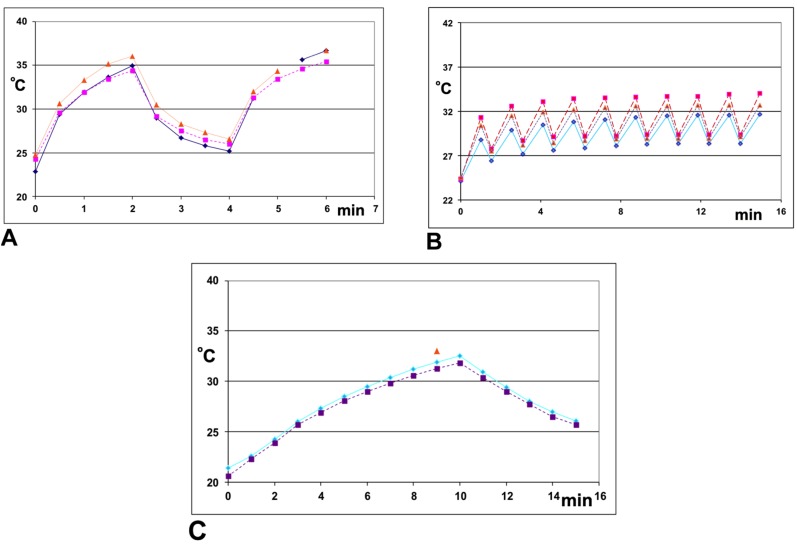
Temperature profiles of 100 μL UD buffer in 96 well microtiter plates incubated using **A**—250 W of microwave irradiation according to a pulse pattern of 2 min_on_ − 2 min_off_ − 2 min_on_, monitoring wells H1 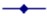, D1 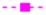, and D5 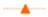; **B**—250 W of microwave irradiation employing a pulse pattern of (1 min_on_ − 33 s_off_)_9_ − 1 min_on_, totaling 10 min of irradiation and 5 min no irradiation; all three replicates monitored well H3; **C**—10 min of incubation at 37 °C followed by five min at 20 °C (no microwave irradiation), monitoring wells C1 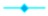, H3 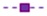, and D9 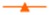. The temperature inside the microwave was monitored using a polytetrafluoroethylene (PTFE) coated fiber optic probe connected to the PELCO BioWave Pro^®^. Temperature inside the wells of the microplate placed inside the 37 °C incubator was followed using an 800012, Type K, Dual Channel Digital Thermometer (SPER Scientific, Ltd., Scottsdale, AZ, USA).

Figure 1 compares the temperature profiles of microtiter plates exposed to microwave radiation (2.45 GHz) *vs.* when placed in a 37 °C incubator. The 2 min_on_ − 2 min_off_ − 2 min_on_ pulse pattern (2–2–2, Figure 1A) entails a total of 4 min of irradiation and a total incubation time of six minutes with the wells reaching a maximum temperature of 36 °C with 250 W of irradiation. The (1 min_on_ − 33 s_ff_)_9_ − 1 min_on_ pulse pattern (1–33, Figure 1B) provides a total of 10 min of irradiation and five minutes of cooling for a total incubation time of 15 min. The 1–33 pulse pattern with 250 W displayed an average peak temperature of 32.1 ± 0.8 °C cooling to 28.1 ± 1.4 °C between microwave pulses. A similar pattern was observed using 350 W, though the average maximum temperature was 35.6 ± 1.5 °C, with some cycles exceeding 40 °C, and an average cooling temperature of 29.7 ± 2.1 °C. The temperature profile for a microtiter plate placed inside a 37 °C incubator for 10 min followed by five min on a lab bench at 20 °C (10–5, Figure 1C), reached a maximum temperature of 33 °C and cooled to 26 °C.

Table 1 summarizes the performance of the ricin ELISA using different pulse patterns for ricin in UD (diluted milk) buffer. The 2–2–2 pulse pattern at 250 W provided a significant improvement in the LOD to 2.9 ng/mL from 4.5 ng/mL observed for samples not irradiated for the six minutes of incubation. In contrast, 250 W applied using the 1–33 pulse pattern generated LOD values comparable to incubating for 1 h at 37 °C. This probably was due to heating since comparable LOD values were obtained when the plates were incubated for 10 min at 37 °C. The five min at 20 °C following incubation at 37 °C did not improve the LOD value but did substantially increase the measured response, potentially important towards the assay’s ruggedness.

**Table 1:** Effect of Microwave Irradiation on Ricin ELISA

Incubation ^a^	Wattage ^b^	Solvent ^c^	LOD ^d^	Response ^e^ 25 ng/mL	Bkgd
2 min_on_ − 2 min_off_ − 2 min_on_	0	UD	4.6 ± 1.4 ^f^	0.046 ± 0.01	0.07
2 min_on_ − 2 min_off_ − 2 min_on_	250	UD	2.9 ± 1.2	0.08 ± 0.05	0.07
(1 min_on_ − 33 s_off_) 9 − 1 min_on_	250	UD	0.6 ± 0.4	0.25 ± 0.05	0.08
(1 min_on_ − 33 s_off_) 9 − 1 min_on_	300	UD	0.38 ± 0.16	0.29 ± 0.03	0.08
10 min 37 °C		UD	0.45 ± 0.05	0.21 ± 0.01	0.07
10 min 37 °C − 5 min Rm T		UD	0.33 ± 0.04	0.34 ± 0.04	0.08
1 h 37 °C		UD	0.4 ± 0.1	1.36 ± 0.15	0.13

^a^ Incubation of ricin, detector, and conjugate as described below. Substrate incubated at room temp for 30 min; ^b^ Wattage of microwave irradiation applied; ^c^ UD buffer consisted of 105 mM NaPi/75 mM NaCl/2.5% NFDM/0.05% Tween-20, pH 6.8; ^d^ Limit of detection: Concentration that generated response equivalent to background plus 3-times the standard deviation (SD); ^e^ Response (OD_410nm_) generated by ricin at 25 ng/mL after subtracting the background (Bkgd) generated by UD buffer; ^f^ Data are the average (±SD) of triplicate analyses except for ‘10 min 37 °C’, which was ran in duplicate (±range/2).

**Figure 2:**
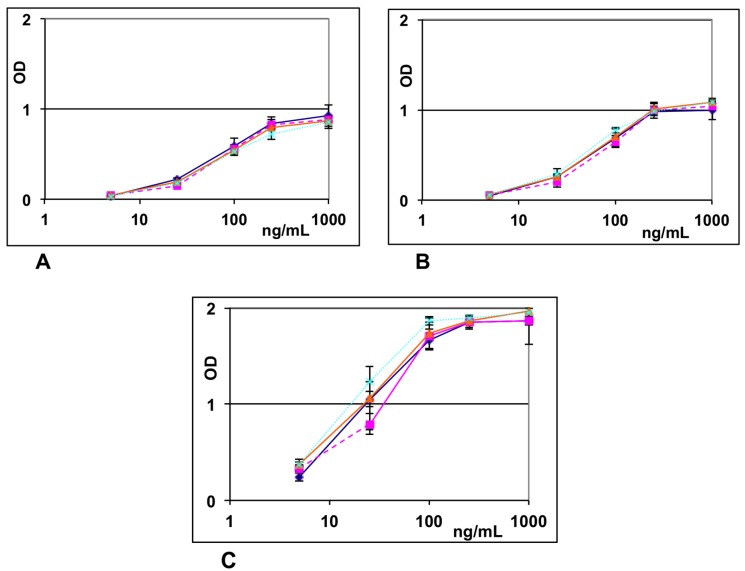
Detection of ricin in spiked foods using **A**—microwave irradiation at 300 W with pulse sequences of (1 min_on_ − 33 s_off_)_9_ − 1 min_on_; **B**—15 min in a 37 °C incubator; or **C**—1 h in a 37 °C incubator. chocolate-hazelnut butter spread 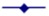, whole wheat pita bread 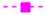, orange juice with pulp 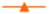, and cola flavored regular (not diet) soda 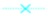. Samples prepared and analyzed in triplicate with the responses converted to concentration based on standards prepared at 0, 0.1, 0.25, 1, 5, 25, 50, 100, 250, 500, and 1000 ng/mL, error bars represent ± one standard deviation.

Figure 2 and Table 2 compare the detection of ricin spiked into chocolate-hazelnut spread, whole wheat pita bread, orange juice with pulp, and cola-flavored regular soda. The 1–33 pulse pattern was not as effective in detecting ricin in whole wheat pita bread, orange juice, and cola as in the chocolate-hazelnut spread (Table 2). The chocolate-hazelnut spread displayed an LOD value comparable to the levels observed in Table 1 with UD buffer (dilute non-fat milk) of 0.4 ng/mL. In contrast, the pita bread, orange juice, and cola displayed LOD values of 2.8, 4.6, and 6.5 ng/mL, respectively, which were characteristic of the LOD value displayed for 6 minutes of incubation (4.5 ng/mL) without any microwave irradiation or heating (Table 1). Interestingly, when incubated for 15 min at 37 °C, the chocolate-hazelnut spread, pita bread, orange juice, and cola displayed LOD values of 0.3, 0.3, 0.5, and 1.6 ng/mL that upon one hour of incubation at 37 °C decreased to ≤0.4 ng/mL. The only difference between the food samples and the UD buffer sample analyses involved the first incubation step, incubation of the food extract, since following the first wash cycle the assays were identical except for the amount of ricin bound to the capture antibodies. Thus, the significantly greater LOD values observed for pita bread, orange juice, and cola when irradiated using the 1–33 pulse pattern *vs.* the LOD values observed with UD buffer (dilute milk) and chocolate-hazelnut spread must be due to the food matrices affecting the efficacy of the microwave irradiation to catalyze the capture of the ricin analyte. The observation of comparable recovery levels makes it unlikely that the problem is due to sequestering by the food matrix. The slightly improved, apparent LOD values for some of the food samples when incubated for 1 h at 37 °C *vs.* the buffer samples may reflect various artifacts of foods and the limited precision of the calibration standards used. Indeed, one potential artifact of foods that is often overlooked is the ability of bread products to increase the analyte concentration by undergoing hydration and reducing the amount of free solvent [19].

**Table 2:** Effects of Incubation on Detection of Ricin Spiked into Food Products.

	Microwave ^a^			15 min 37 °C			1 h 37 °C		
	LOD ^c^ ng/mL	Recovery ^e^ %	Bkgd ^d^ OD_410nm_	LOD ng/mL	Recovery %	Bkgd OD_410nm_	LOD ng/mL	Recovery %	Bkgd OD_410nm_
Chocolate-Hazelnut Spread ^b^	0.4	93 ± 8	0.055 ± 0.001	0.3	81 ± 7	0.058 ± 0.001	0.1	79 ± 10	0.072 ± 0.002
Whole Wheat Pita Bread	2.8	87 ± 19	0.070 ± 0.008	0.3	78 ± 12	0.073 ± 0.001	0.09	80 ± 15	0.117 ± 0.002
Orange Juice with Pulp	4.6	91 ± 6	0.071 ± 0.013	0.5	90 ± 7	0.070 ± 0.002	0.3	89 ± 13	0.106 ± 0.008
Soda (cola)	6.5	83 ± 8	0.093 ± 0.015	1.6	93 ± 4	0.091 ± 0.006	0.4	98 ± 5	0.137 ± 0.009

^a^ All incubation steps consisted of either 300 W of microwave irradiation according to the pulse pattern (1 min_on_ − 33 s_off_) − 1 min_on_ or in an incubator for 15 min at 37 °C, or 1 h at 37 °C; ^b^ Food samples were spiked with ricin to final concentrations of 0, 5,25, 100, 250, and 1000 ng/mL. Samples prepared in triplicate; ^c^ LOD values calculated as the concentration of ricin that generates a response equal to the background plus three-times the standard deviation; ^d^ Overall average percent recovery (±one standard deviation) for triplicate food samples spiked with 5, 25, 100, and 250 ng/g ricin; ^e^ Average background response (OD_410nm_) of the food generated in the ELISA ± one standard deviation.

## 3. Experimental Section

Ten mM PBS (catalog # P3813), Tween-20^®^ (catalog # P7949) and other chemical reagents were purchased from Sigma-Aldrich Chemical Company (St. Louis, MO, USA). Ricin was purchased from Vector Laboratories (Burlingame, CA, USA). Chocolate-hazelnut spread, whole-wheat pita bread, orange juice with pulp, and cola-flavored regular (not diet) soda were purchased from a local retail store.

### 3.1. Instrumentation

A PELCO BioWave Pro^®^ 2.45 GHz microwave processor in conjunction with the ColdSpot Pro^®^ (Ted Pella, Inc. Redding, CA, USA) was used to irradiate ELISA polystyrene, flat bottom microtiter plates containing 100 µL of aqueous sample per well. To minimize variability, all microtiter plates were positioned identically on the ColdSpot Pro^®^, which was maintained at 20 °C using a circulating water bath. Temperature was monitored using a polytetrafluoroethylene (PTFE) coated fiber optic probe placed typically into well H3, unless noted otherwise. Wattage was checked based on the change in temperature of 1 L of water after irradiation for 2 min according to the equation W = ΔT × 35. The measured wattage was within 8% of the target setting, often within 4%.

Temperature in the wells of the microplate when incubated inside the 37 °C incubator were followed using an 800012, Type K, Dual Channel Digital Thermometer (SPER Scientific, Ltd. Scottsdale, AZ, USA). Absorbance measurements were made using an Infinite 200 microplate reader (Tecan, Inc., San Jose, CA, USA).

### 3.2. Gliadin ELISA

The RIDASCREEN Gliadin R5 sandwich ELISA was purchased from R-Biopharm, Inc. (Washington, DC, USA) and used according to manufacturer’s instructions. The standards were supplied as aqueous solutions containing 0, 5, 10, 20, 40, and 80 ng/mL gliadin. The assay consisted of incubating the sample (or gliadin standard solutions), enzyme-conjugate, and substrate for 30 min at room temperature, with the last incubation being performed in the dark. The absorbance was measured at 450 nm after adding acid to stop color development. All samples were prepared and analyzed in triplicate.

### 3.3. Ricin ELISA

Ricin ELISA test kits were acquired from Tetracore^®^, Inc. (cat # TC-4001-002, Rockville, MD, USA). The microtiter plates were coated with capture antibody and blocked using UD buffer (105 mM NaPi/75 mM NaCl/2.5% non-fat milk/0.05% Tween-20, pH 6.8) as recommended by the manufacturer. Ricin standards were prepared in PBS or UD buffer at 0, 0.1, 0.25, 1, 5, 25, 50, 100, 250, 500, and 1000 ng/mL. UD buffer, strongly buffered dilute milk, has been shown to prevent non-specific binding of lectins that may be present in foods [21] and is sufficiently buffered to handle highly acidic foods.

Pita bread, chocolate-hazelnut spread, orange juice with pulp, and cola-flavored regular (not diet) soda were spiked with ricin at 0, 5, 25, 100, 250, and 1000 ng/g (ng/mL for beverages, ppb) and allowed to sit at room temperature for one hour prior to analysis. To minimize differences between the spiked samples, the ricin was added from multiple stock solutions, freshly prepared in PBS at 12 to 50-times the final concentration. Sample preparation for ELISA analysis consisted of mixing 0.5 g of the spiked chocolate-hazelnut spread with 19.5 mL UD buffer. The spiked pita bread, orange juice, and soda were mixed at 0.1 g (mL for the beverages) with 3.9 mL UD buffer. After extensive vortexing, the samples were allowed to sit for 15 min to allow the settling of insoluble material before pipetting using wide bore tips. To minimize differences in incubation times of the various samples, a polypropylene 2 mL well microtiter plate was used as a template from which 100 µL samples were transferred to the (capture antibody coated, UD buffer blocked) ELISA plate using a 12-channel pipettor. The sample, primary detector antibody, and secondary detector-conjugate incubation steps were performed identically within an assay; whether entailing incubation at 37 °C, 20 °C, or with microwave irradiation. The primary detector antibody and secondary detector-conjugate (to horse radish peroxidase, HRP) were prepared at 55 µL and 2.2 µL per 11 mL UD buffer, respectively. In all cases, the substrate was incubated for 30 min at 20 °C with no exposure to light prior to reading the absorbance at 410 nm. Since, the ELISA test kit is also supplied with the microtiter plates precoated with capture antibody (TC-4002-002 and TC-4004-001), the throughput of the ELISA is described as 5 h, from preparation of the samples through analysis. All samples were prepared and analyzed in triplicate.

### 3.4. ELISA data analysis

LOD values were calculated as the concentration of analyte necessary to generate an absorbance equal to the background plus three-times the standard deviation.

Recovery was calculated as the amount of detected analyte *vs.* the amount spiked into a food sample. To eliminate analytical errors associated with heterogeneous distribution of an analyte and to focus on the effects of the food matrix on assay performance, the analyte was spiked into samples that were completely extracted during sample preparation. Calibration curves were generated using pure analyte in buffer with all samples and standards ran in triplicate.

## 4. Conclusions

The various microwave pulse patterns (2–2–2 and 1–33) and the abbreviated incubations reduced the time it took to complete the ricin ELISA, including sample preparation, from approximately five hours to less than two. Though the differences in performance were significant, under all conditions the ELISA displayed an LOD of < 10 ng/mL and displayed recoveries ranging from 78% to 98%, sufficient to meet all analytical needs (Table 2) and below levels considered important in a recent multi-laboratory validation of handheld devices for the detection of ricin [22]. It is important to note that the average percent recoveries were for the food samples spiked with 5, 25, 100, and 250 ng/mL ricin and did not include the 1000 ng/mL samples which typically displayed the onset of a saturation response and would be unreliable in such calculations.

The data do not completely rule out a non-thermal enhancement by microwave irradiation on ELISA performance. However, the enhancement observed could be approximated by thermal incubation, indicating that a non-thermal effect targeting macromolecules may be negligible. Further, the observation that some food extracts performing more poorly with microwave irradiation raised questions about the utility of microwave irradiation as a universal method to enhance macromolecular detection in food. Microwave irradiation may provide an effective method of heating some samples quickly (Figure 1A,B) with preliminary studies indicating further improvements in assay performance for some samples at higher levels of irradiation that resulted in temperatures reaching 50 °C. However, differences between foods and the effects of heating make such an approach unpredictable without first validating performance with each specific food.
